# Five ways to get a grip on grouped self-assessments of competence for program evaluation

**DOI:** 10.36834/cmej.69276

**Published:** 2020-08-06

**Authors:** Rebecca Zhao, Marcel D’Eon

**Affiliations:** 1 University of Saskatchewan

## Abstract

Self-assessments conducted by individuals when taken together (grouped) provide valid and accurate measures of learning outcomes of the group. This is useful for program evaluation. Grouped self-assessments are simple to understand and construct, easy to implement, relatively accurate, and do not require extensive and complex pre-post testing measures. However, group self-assessments have the potential to be misused. To examine how group self-assessments have been used in medical education, we conducted a search of journal articles published in 2017 and 2018 from eight prominent medical education journals. Twenty-seven (*n* = 27) articles that used self-assessments for program evaluation were selected for data extraction and analysis. We found three main areas where misuse of self-assessments may have resulted in inaccurate measures of learning outcomes: measures of “confidence” or “comfort”, pre-post self-assessments, and the use of ambiguous learning objectives. To prevent future misuse and to build towards more valid and reliable data for program evaluations, we present the following recommendations: measure competence instead of confidence or comfort; use pre-test self-assessments for instructional purposes only (and not for data); ask participants to do the post-intervention self-assessments first followed by retrospective pre-intervention self-assessments afterwards; and use observable, clear, specific learning objectives in the educational intervention that can then be used to create the self-assessment statements.

## Abstract (fr)

Les auto-évaluations effectuées individuellement, quand mises en commun(regroupées) fournissent des mesures valides et précises des résultats de l’apprentissage du groupe. Ceci s’avère utile dans le cadre d’évaluation de programme. Les auto-évaluations groupées sont faciles à comprendre, à créer et à mettre en œuvre. De plus, elles sont relativement précises et ne requièrent pas de mesures exhaustives et complexes avant et après l’évaluation. Cependant, il est possible de mal utiliser les auto-évaluations de groupe. Afin d’examiner de quelle façon les auto-évaluations de groupe ont été utilisées dans la formation médicale, nous avons dépouillé des articles de huit revues médicales importantes publiées en 2017 et 2018. Vingt-sept (n=27) articles ayant utilisé les auto-évaluations à des fins d’évaluation de programme ont été sélectionnés pour être analysées. Nous avons trouvé trois domaines principaux où la mauvaise utilisation des auto-évaluations a peut-être occasionné des mesures inexactes des résultats de l’apprentissage : mesures de « confiance » ou d’« aisance », auto-évaluations pré/post et l’emploi d’objectifs d’apprentissage ambigus. Afin de prévenir l’utilisation future incorrecte et de miser sur des données plus valides et fiables à des fins d’évaluation de programme, nous présentons les recommandations suivantes : mesurer la compétence au lieu de la confiance ou l’aisance; utiliser les auto-évaluations prétest à des fins d’enseignement seulement (et non comme données); demander aux participants de faire les auto-évaluations post-intervention d’abord suivies par les auto-évaluations préintervention rétrospectives après; utiliser des objectifs d’apprentissage observables, clairs et spécifiques lors de l’intervention de formation qui peuvent servir de base aux items d’auto-évaluation.

Grouped self-assessments (GS-a) are accurate and valid measures of learning outcomes for the target group in the context of program evaluation.^1^ We use the definition of program evaluation by the Canadian Evaluation Society found on their website (https://evaluationcanada.ca/what-is-evaluation): evaluation is the systematic assessment of the design, implementation or results of an initiative for the purposes of learning or decision-making. GS-a, therefore, systematically collect data that can help determine some of the results of a learning initiative. By self-assessment, we mean the rating of one’s own ability to meet a learning objective or to performs a task. For example, people may be asked to rate the extent to which they are able to describe the progression of Type 2 diabetes in a middle-aged patient. These individual self-assessments do not predict individual achievement but when grouped give a valid and reliable measure of how much the group as a whole learned from the intervention.^1^ GS-a is the arithmetic mean of all the self-assessments completed by members of the target audience, usually a group of learners. Furthermore, GS-a are simple to construct (they are based on the objectives of the educational intervention) and easy to administer (both the pre- and post-intervention self-assessments are collected after the intervention).^2^ However, these data collection methods could be and are misused in program evaluations. In this article, we describe how some researchers use and misuse GS-a in health professions education and explain why some of these may yield poor data for program evaluation.

To provide empirical evidence for the uses and especially misuses of self-assessments, we conducted a quick search for articles published in 2017 and 2018 in eight medical education journals: Academic Medicine, Advances in Health Sciences Education, BMC Medical Education, Canadian Medical Education Journal, Medical Education, Medical Teacher, and Perspectives on Medical Education. Twenty-seven articles that used GS-a for program evaluation were selected for data extraction and analysis. We tallied the number of times that these features of self-assessment were used: use of grouped pre self-assessment as data; measures of comfort or confidence, competence, and readiness; comparisons of pre-test and post-test data; comparisons of retrospective pre-test and post-test data, analyses of post-test data only; and the presence of observable, clear, specific learning objectives. See Table 1.

**Table 1:** Summary of features found in grouped self-assessment studies.

Authors (Year)	Used pre-Self-assessment as data	Measured confidence and/or comfort	Measured competency	Measured readiness	Pre- vs true post-test comparison	Retrospective pre- vs post-test comparison	Post-test only	Observable, clear, specific learning objectives
Total: 27	19	18	6	2	19	4	5	22
Bartlett, et al. (2017)	Yes	Yes	No	No	Yes	No	No	Yes
Bartman, et a. (2018)	No	No	Yes	No	No	Yes	No	Yes
Chokshi, et al. (2017)	No	No	No	No	No	Yes	No	No
Clay, et al. (2017)	Yes	Yes	No	No	Yes	No	No	Yes
Clementz, et al. (2017)	No	Yes	No	No	No	No	Yes	Yes
Držaić, et al. (2018)	No	No	Yes	No	No	No	Yes	Yes
Garibaldi, et al. (2017)	Yes	Yes	No	No	Yes	No	No	Yes
Gomes, et al. (2017)	No	No	No	No	No	No	Yes	Yes
Kaminetzky, et al. (2017)	No	Yes	Yes	No	No	No	Yes	Yes
Lévesque, et al. (2018)	Yes	No	Yes	No	Yes	No	No	Yes
Levine, et al. (2018)	Yes	Yes	No	No	Yes	No	No	Yes
Loke, et al. (2017)	No	Yes	No	No	No	Yes	No	No
Ludwig, et al. (2017)	Yes	Yes	No	No	Yes	No	No	Yes
O’Donoghue, et al. (2018)	Yes	Yes	No	No	No	No	Yes	Yes
Peluso, et al. (2017)	Yes	Yes	No	Yes	Yes	No	No	Yes
Pettignano, et al. (2017)	Yes	No	No	No	Yes	No	No	No
Phillips, et al. (2017)	Yes	Yes	No	Yes	Yes	No	No	Yes
Rassbach, et al. (2018)	Yes	Yes	No	No	Yes	Yes	No	No
Richardson, et al. (2018)	Yes	Yes	No	No	Yes	No	No	Yes
Rusiecki, et al. (2018)	Yes	Yes	No	No	Yes	No	No	Yes
Sabouni, et al. (2017)	Yes	Yes	No	No	Yes	No	No	Yes
Shiels, et al. (2017)	No	Yes	Yes	No	Yes	No	No	Yes
Sopoaga, et al. (2017)	Yes	No	No	No	Yes	No	No	Yes
Tchekmedyian, et al. (2017)	Yes	No	Yes	No	Yes	No	No	Yes
Wilkes, et al. (2017)	Yes	Yes	No	No	Yes	No	No	Yes
Yang, et al. (2017)	Yes	No	No	No	Yes	No	No	No
Yeung, et al. (2017)	Yes	Yes	No	No	Yes	No	No	Yes

Some studies used a topic or domain area rather than clearly written and observable learning objectives connected to the intervention. Specific observable statements of learning outcomes will give more accurate data than broad, vague statements that might be open to great variation in interpretation.

Many studies we found used “confidence” and “competence” interchangeably. Confidence suggests one’s willingness to undertake an activity and to continue undertaking the task if initially unsuccessful, whereas competence alludes to what is known about one’s own ability and one’s previous experience of carrying out a task.^3^ They asked questions such as “how comfortable are you…?” Of the 27 selected reports, 18 studies measured perceived confidence in or comfort with performing certain tasks.^4^^–^^21^ Six studies measured competency or ability,^10^^,^^13^^,^^22^^–^^25^ two studies measured readiness,^9^^,^^16^ and five studies lacked sufficient detail to determine what they used.^26^^–^^30^ Measuring one’s willingness to perform a task or one’s confidence helps promote reflection of performance^3^ but is not the most effective method of evaluating learning or performance.

Additionally, pre-test GS-a coupled with post-test GS-a are inadequate measurements of program results.^31^^,^^32^ Pre-post measures are prone to response shift bias in subjects’ estimates of training effects.^31^ Specifically, an intervention may change one’s standard of evaluation because of changes in the participant’s internal standards, values, or understandings.^33^ Thus, changes in a respondent’s self-evaluation standard would be reflected in their post-test self-assessment. According to our analysis, five studies had not used any pre-intervention assessments at all.^5^^,^^10^^,^^18^^,^^22^^,^^28^ Eighteen studies administered pre-post self-assessments,^4^^,^^6^^–^^9^^,^^12^^–^^17^^,^^19^^,^^21^^,^^23^^,^^25^^,^^27^^,^^29^^,^^30^ whereas only three studies employed post-intervention retrospective pre-test self-assessments.^11^^,^^24^^,^^26^ One study had employed both pre-intervention pre-test and post-intervention retrospective pre-test self-assessments.^20^ The majority of selected studies in our analyses may have been affected by the response shift bias with pre-post self-assessments, suggesting inaccurate data for program evaluation.

Learning is an important purpose of all educational interventions. Thus, one should be referring to learning objectives to analyze or judge educational interventions. Some studies used a topic or domain area rather than clearly written and observable learning objectives tied to the intervention. Specific observable statements of learning outcomes will give more accurate data than broad, vague statements that might be open to great variation in interpretation. Of the 27 selected studies, 22 studies used the observable, clearly written, specific learning objectives from the program or intervention in their self-assessments.^4^^–^^10^^,^^12^^–^^18^^,^^21^^–^^25^^,^^28^^,^^29^ Yet, five studies either lacked sufficient details to determine what they used or used topic areas and broad domains instead of learning objectives.^11^^,^^20^^,^^26^^,^^27^^,^^30^ When self-assessments refer to ambiguous learning objectives, respondents may not be able to accurately interpret what they are expected to assess. If ambiguous learning objectives or broad topic areas are used, respondents’ interpretations of what they were supposed to self-assess may differ from the interpretations of other respondents, the educators, and researchers.^34^

## How to Use Grouped Self-Assessments in Program Evaluation

**Figure 1:**
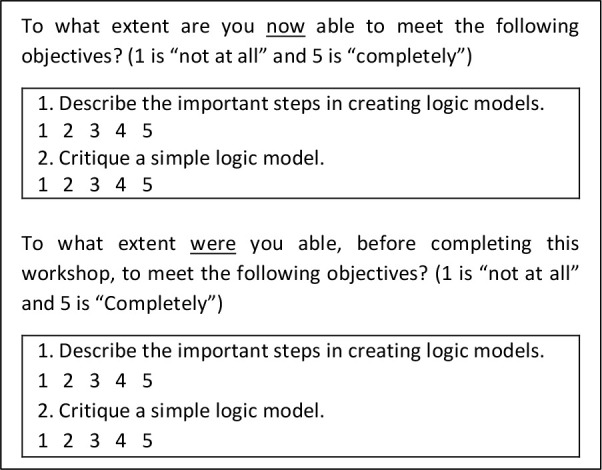
An example of a post-test self-assessment and a retrospective pre-test self-assessment at the end of an educational session on creating logic models for program evaluation.

1. **Use competence as a criterion for measurement; not confidence or comfort**. There is a difference between measuring competence versus confidence or comfort. The former refers to perceived ability to do an activity (as defined by the objectives of the educational initiative), whereas the latter refers to one’s judgement of their willingness to do an activity. Thus, we should ask people to assess their own competence or ability to do a task and not their confidence or comfort in doing that task. We might ask, then, to what extent the learners are able to accomplish a task or can describe a phenomenon not how comfortable or confident they are in performing the task or describing the phenomenon
2. **Have observable, clearly written, specific learning objectives in the self-assessments**. When creating self-assessments, one should avoid ambiguous questions so that all respondents may interpret questions similarly. Additionally, self-assessment questions or prompts should be specific enough that respondents know what their response should be about, and researchers know what it means.^35^
3. **Use pre-test self-assessments for instructional purposes only**. Asking learners to assess themselves before the educational intervention alerts them to the goals of instruction and prepares them for learning. This can improve the effectiveness of the intervention but not the evaluation. Subject to the response shift bias explained above, pre-test self-assessments alone or coupled with post-test self-assessments will yield inaccurate data. Clearly, the standards people use to self-assess change as they learn.
4. **Use post-intervention retrospective pre-test self-assessments with post-intervention self-assessments**. A pre-test gathers data before the intervention. A retrospective pre-test gathers data from before the intervention (“pre”) but by looking back (retrospectively) after the intervention. This type of assessment will minimize the risk of the response shift bias in influencing the accuracy of the self-assessments. In the course of the educational intervention, a respondent will change the standard against which they would assess themselves. Once they learn more, their standard goes up. Hence, as explained earlier, it is better to ask participants after the intervention, the extent to which they were able to describe the progression of diabetes in a middle-aged man before the session took place.
5. **Following the educational intervention, ask first about their current condition (post-intervention) and then ask respondents to look back to just before they started their educational sessions**.^32^ It is best to ask for the entire set of post-intervention self-assessments first followed by the retrospective pre-test self-assessments. Do not ask for self-assessments, both pre- and post- objective by objective, as respondents may complete the questions to obtain the result according to how much they believe they have learned. We provide an example in Figure 1.

To avoid the dangerous black ice of grouped self-assessments, use our five guidelines to get a grip on your next program evaluation We have created an example self-assessment for the end of an educational session on creating logic models for program evaluation. See Figure 1. Finally, we hope that researchers will further explore Gs-a in the search for other “better” practices and for the mechanisms that make them work.
